# Multifunctions of CRIF1 in cancers and mitochondrial dysfunction

**DOI:** 10.3389/fonc.2022.1009948

**Published:** 2022-10-03

**Authors:** Yangzhou Jiang, Yang Xiang, Chuanchuan Lin, Weiwei Zhang, Zhenxing Yang, Lixin Xiang, Yanni Xiao, Li Chen, Qian Ran, Zhongjun Li

**Affiliations:** ^1^ Laboratory of Radiation Biology, Laboratory Medicine Center, Department of Blood Transfusion, The Second Affiliated Hospital, Army Military Medical University, Chongqing, China; ^2^ State Key Laboratory of Trauma, Burn and Combined Injuries, The Second Affiliated Hospital, Army Medical University, Chongqing, China

**Keywords:** CR6-interacting factor 1, cell cycle regulation, cell proliferation regulation, cancer treatment, mitochondrial ribosomal protein, mitochondrial dysfunction

## Abstract

Sustaining proliferative signaling and enabling replicative immortality are two important hallmarks of cancer. The complex of cyclin-dependent kinase (CDK) and its cyclin plays a decisive role in the transformation of the cell cycle and is also critical in the initiation and progression of cancer. CRIF1, a multifunctional factor, plays a pivotal role in a series of cell biological progresses such as cell cycle, cell proliferation, and energy metabolism. CRIF1 is best known as a negative regulator of the cell cycle, on account of directly binding to Gadd45 family proteins or CDK2. In addition, CRIF1 acts as a regulator of several transcription factors such as Nur77 and STAT3 and partly determines the proliferation of cancer cells. Many studies showed that the expression of CRIF1 is significantly altered in cancers and potentially regarded as a tumor suppressor. This suggests that targeting CRIF1 would enhance the selectivity and sensitivity of cancer treatment. Moreover, CRIF1 might be an indispensable part of mitoribosome and is involved in the regulation of OXPHOS capacity. Further, CRIF1 is thought to be a novel target for the underlying mechanism of diseases with mitochondrial dysfunctions. In summary, this review would conclude the latest aspects of studies about CRIF1 in cancers and mitochondria-related diseases, shed new light on targeted therapy, and provide a more comprehensive holistic view.

## Introduction

**Figure 1:**
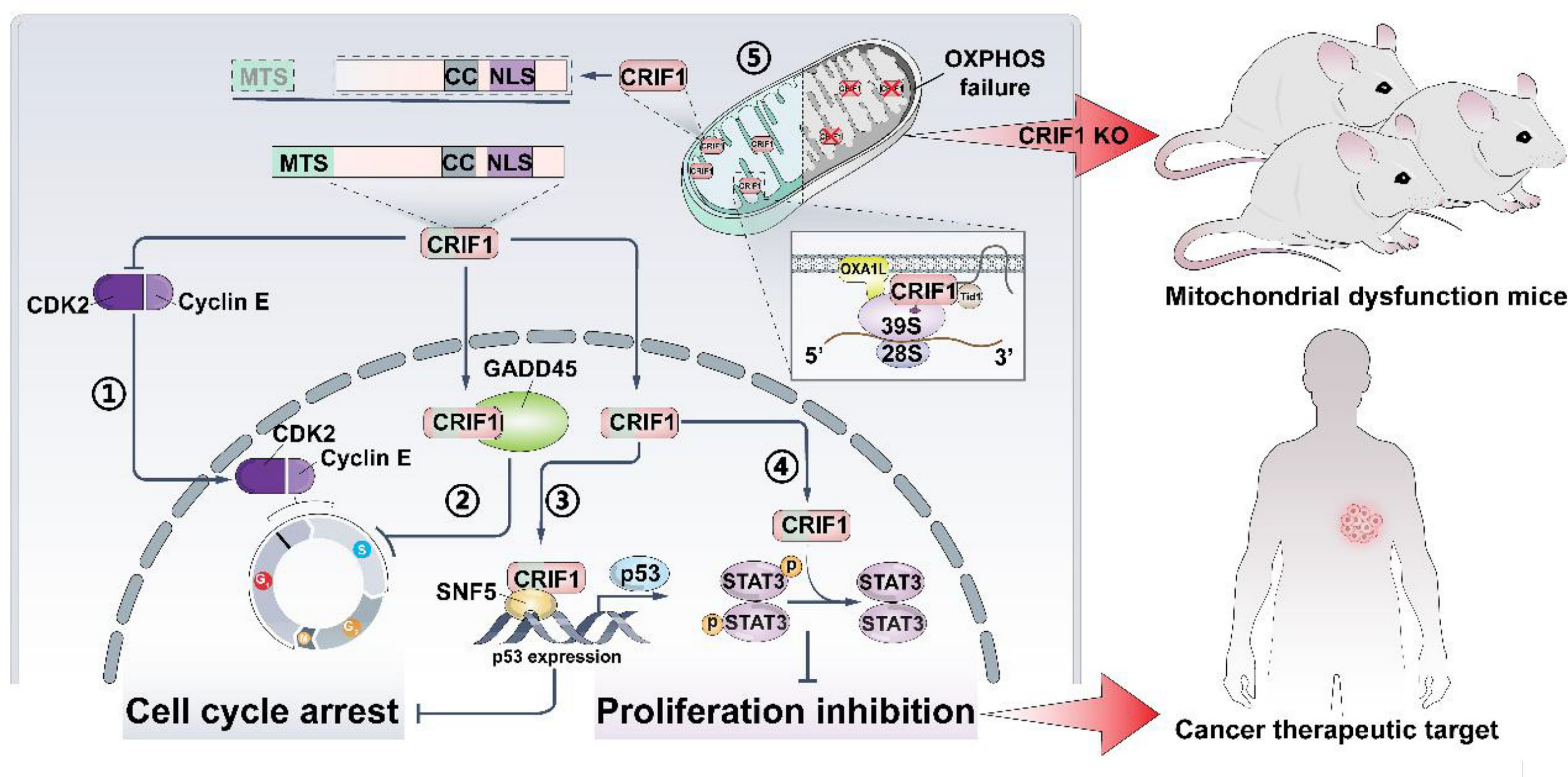
CRIF1 regulates cell cycle, cell proliferation, and OXPHOS capacity. It could be a cancer therapeutic target, and knocking down CRIF1 is an effective way to establish mitochondrial dysfunction in mice. CRIF1 binds to CDK2 and inhibits its activity ①. CRIF1 binds to Gadd45 family proteins and enhances its activity ②. CRIF1 increases P53 expression by coactivating with SNF5 ③. CRIF1 decreases STAT3 phosphorylation and inhibits cell proliferation ④. CRIF1 transports into the mitochondrial matrix, associates with the LSU of the mitoribosome, and regulates OXPHOS capacity ⑤.

As an irreversible progress, the cell cycle balance of normal cells mainly depends on regulating the activities of cyclin-dependent kinases (CDKs). Cancer cells exhibit uncontrolled cell cycle progression and mitosis, due to the extraordinary proliferative ability. To sustain the capacity of rapid proliferation, cancer cells are peculiarly prone to get energy from glycolysis and display enhanced aerobic glycolysis which are referred to as the ‘Warburg effect’ (1). Researchers intend to exploit new therapeutic strategies or targets following the above two aspects to overcome the resistance encountered in cancer therapy. One strategy is to deepen the cell cycle exit, and the other one is to interfere the energy metabolism of cancer cells. However, the initial CDK inhibitors show obvious toxicities and limited efficacy, and the new class inhibitors (e.g., palbociclib, ribociclib, and abemaciclib) also display obvious tolerability (2). Recently, a novel factor (**Figure 1**), CR6-interacting factor 1 (CRIF1), was discovered to regulate the cell cycle progression. CRIF1 was also found to be expressed in mitochondria and to influence the OXPHOS capacity. All these suggest that CRIF1 has a considerable potential of being a new target for regulating the cell cycle and cell energy metabolism and might be applied in cancer treatment.

The CRIF1 gene, which is located on chromosome 19p13.2 on the human reference genome and is known as GADD45GIP1, CKBBP2, and CKbetaBP2, holds a 669-bp open reading frame (ORF) encoding a protein of 222 amino acids. It is ubiquitously expressed in mammalian tissues, with high abundance in the heart, lymph nodes, trachea, thyroid gland, and adrenal tissues (3). In posttranslational modification of CRIF1, it is phosphorylated on serine residue at position 221 by interacting with the β subunit of CKII and promotes proliferation of cells (4). CRIF1 was originally identified as a nuclear protein which enhances the activity of Gadd45 family proteins. As the underlying role of CRIF1 further develops, it is believed that CRIF1 plays a pivotal role in cell cycle progression including the induction of cell cycle arrest through three main ways (3, 5–9). First, CRIF1 binds to the Gadd45 family proteins and consequently inhibits the activities of Cdc2. Second, CRIF1 can directly interact with CDK2 *via* the long alpha helical region and induces cell cycle arrest. Third, CRIF1 enhances the expression of P53 and its downstream genes including P21, which mediates P53-induced G1/S arrest. Overall, CRIF1 is an important regulator of the cell cycle and is a potential tumor suppressor. In the cancer field, it is critical to enhance the sensitivity of cancer cells to treatments (radiotherapy and drugs), while CRIF1 could be a target to turn the cancer cells resistant to sensitive and could become a novel therapeutic target due to its functions in the cell cycle.

Mitochondria are well-known organelles which provide ATP *via* OXPHOS for cells. However, cancer cells are prone to obtain energy by glycolysis instead of aerobic oxidation, indicating that metabolic rewiring is important in malignant transformation for cancer cells (10). Beyond energy supplies, mitochondria are central players of metabolic signaling and are potential energy sensors and beacons (11). The mitochondrial proteins are partly encoded by their own DNA (mtDNA). In mammals, mtDNA encodes 13 essential proteins which are crucial for OXPHOS (12, 13). Although CRIF1 is not encoded by mtDNA, it is identified as a mitochondrial ribosomal protein (MRP) in the large subunit (LSU) (14). The expression of CRIF1 directly regulates mitochondrial OXPHOS capacity. Mechanically, CRIF1 is a key protein in the process of integrating OXPHOS polypeptides into the mitochondrial membrane in mammals (15). In different types of human diseases, mitochondrial dysfunction characterized by impaired OXPHOS capacity is implicated in the pathological process. Consequently, to reveal the underlying mechanisms of mitochondrial dysfunction in diseases, CRIF1 is an effective target to regulate the OXPHOS capacity.

## CRIF1, a novel target in cancer treatment

### CRIF1 regulates the cell cycle

The transcription factor P53 and the CDK–cyclin complex are two crucial factors to rule the cell cycle. P53 is a well-known tumor suppressor that upregulates numerous genes resulting in cell cycle arrest, such as Gadd45 and P21. The CDK–cyclin complex determines the phosphorylation of retinoblastoma (Rb) proteins. Phosphorylated Rb (pRb) can dissociate from the Rb–E2F complex and free E2F to the nucleus and contact the E2F transcription factor binding sites in the gene promoters to promote the cell cycle progression (16). The cell cycle machinery proteins are canonical targets in cancer therapy, and many kinase inhibitors have been investigated in preclinical/clinical trial studies or applied in clinical use, especially inhibitors for CDKs (17–20). However, with the increase in clinical application, CDK inhibitors show limitations such as drug resistance, off-target effect, and minimal effects on the kinase-independent role of CDK targets. Beyond ameliorating the structure of inhibitors, researchers attempt to overcome these shortcomings by combining inhibitors with other drugs, selecting patients with identified biomarkers or optimizing treatment sequencing (21–23). Besides, CRIF1 is another protein found to directly bind to Gadd45 and CDK2. This provides a new strategy to interfere with the activity of critical enzymes in the cell cycle; thus, CRIF1 has a considerable potential to become a therapeutic target.

CRIF1 was initially reported as a nuclear protein which can interact with Gadd45 family proteins including Gadd45α, MyD118/Gadd45β, and CR6/OIG37/Gadd45γ (3). Gadd45 family proteins have been implicated in the responses of cell cycle regulation, genomic stability, and apoptosis (24–26). In the cell cycle, Gadd45 family proteins regulate G1/S and G2/M checkpoints in different cell types. Cdc2 is a downstream gene regulated by the Rb–E2F complex. Gadd45 family proteins bind to Cdc2 kinase; at the same time, CRIF1 promotes the inhibiting effect on Cdc2 kinase in an additive manner by directly interacting with Gadd45 proteins (27–30). Overexpressed CRIF1 binds to Gadd45 and suppresses the activity of some critical enzymes in the cell cycle. The percentage of G1 cells is significantly increased (3). Gadd45 family genes were always downregulated in cancers due to promoter methylation (31). Downregulated Gadd45α promoted mammary tumor formation which is driven by Ras activation (32). Further studies revealed that Gadd45 may play different roles depending on oncogenic stress. It was regarded as a prognostic marker in breast cancer and was considered as a target in breast cancer therapy (33–35). Beyond upregulating the expression of Gadd45 family genes, increasing the CRIF1 expression is a new strategy to enhance the capacity of Gadd45 family proteins in the cell cycle. Consequently, the CRIF1 potential becomes a therapeutic target that exercises cell cycle-regulating functions through directly binding to Gadd45.

Further studies found that CRIF1 interacts with CDK2 in both nucleus and cytoplasm by co-immunoprecipitation in Jurkat cells (7). In dividing cells, CDK2 is essential for G1/S transitions and S-phase progression. CDK2 is also driven by various oncogenic signaling pathways to regulate cancers, since potent oncogenes partly enhance the activity of CDK2. Specifically inhibiting CDK2 leads to significant defects in anchorage-independent growth of cancer cells and cells with mutations in oncogenes (36). The activity of CDK2 is inhibited by CRIF1, and cells are arrested in the G0/G1 phase. In line with this, Ran et al. revealed that BMMSCs regulate the cell cycle of leukemic cells by altering the CRIF1 expression. In cocultured leukemic cells, CRIF1 directly interacts with CDK2 and acts as an inhibitor to promote leukemic cells to be arrested in the G0/G1 phase (5, 7). The long alpha helical regions of CRIF1 which include His120, Glu116, and Gln112 were considered to interact with Arg200, Arg214, and Asp210 of CDK2 to form the interaction interface of CRIF1–CDK2. More interestingly, they found that interface inhibitors observably increase ionizing radiation (IR) inhibition potential from 19.9% to 59.6% (37). In cancer treatment, CDK2 inhibitors always show exceptional anticancer activity. CDK2 inhibitors have been screened for decades, and a large number of inhibitors have been identified. Dozens of inhibitors have undergone preclinical studies, and some have been approved for clinical use (38–40). The original CDK2 inhibitors were limited by its off-target effects and high toxicity. Although some improved small-molecule CDK2 inhibitors have progressed to clinical trials or clinical use, their low selectivity is the biggest obstacle encountered in application (41, 42). Different from classical inhibitors targeting the ATP-binding site, these interface inhibitors of CRIF1–CDK2 directly bind to CRIF1 and lead to CDK2 overactivation. Overactivated CDK2 selectively promotes apoptosis and G2/M arrest in osteosarcoma (OS) cell lines (37). In other words, disrupting the complex formation of CRIF1–CDK2 by targeting CRIF1 instead of CDK2 might be a novel strategy to be used for overcoming selectivity issues and regulation of cancer cell radiosensitivity.

CRIF1 also regulates the cell cycle by altering the P53 expression. P53-P21-DREAM-E2F/CHR is an important pathway identified to induce cell cycle arrest. P21 is an important transcriptional target of P53, since it mediates P53-induced G1/S arrest by forming complexes with Cdc2, CDK2, CDK3, CDK4, and CDK6 together with specific cyclins (43). The p53 mutation is always found in ~60% of colorectal cancers. Moreover, mutated p53 activates NF-κB and promotes epithelial–mesenchymal transition (EMT) (44). Researchers found that CRIF1 enhances the expression of P53 and induces G1/S arrest in HCT116 cells. Mechanically, CRIF1 associates with chromatin remodeler SNF5 to bind to the upstream promoter sequences of the p53 gene, increasing the expression of the P53 gene. Downstream target genes such as p21 and Gadd45 are activated. P21 governs Rb phosphorylation and ultimately induces G1/S arrest in HCT116 cells (9). These advances suggest that the regulation of the cell cycle must be a result of various factors, and CRIF1 is probably involved in this progress and acts as a regulator. Depending on cancer type and stage, CRIF1 perhaps exercises its functions *via* different signal pathways which might exhibit different effects on tumorigenesis and progression. Due to the role of CRIF1 in cell cycle regulation, it is expected to become a target for cancer therapy and to enhance the sensitization of radiotherapy and chemotherapy.

### CRIF1 regulates cell proliferation

The high capacity of proliferation is a critical characteristic of cancer cells (45). Therefore, exploiting drugs to kill cancer cells with high basal level of proliferation and regeneration is another common strategy in cancer treatment. Clinically available targeted therapies always focus on blocking the constitutive activation of signal transduction pathways and regulating hormone or receptor level (46–48). For example, the mitogen-activated protein kinase (MAPK) pathway potentially acts as the most frequently mutated signaling pathway in human cancer and plays an important role in cancer cell proliferation. Targeting the MAPK pathway has led to the clinical success of BRAF and MEK inhibitors, and ERK inhibitors exhibit potential advantages of improving cancer therapy (49). However, due to cancer heterogeneity and genomic instability, these inhibitors exhibiting a high frequency of drug resistance is the biggest obstacle encountered in clinical application. The JAK-STAT pathway is characterized by rapid membrane-to-nucleus signaling transduction, and its aberrant activation is closely associated with tumor formation (50). Moreover, some steroid hormones are regarded as cancer initiators and alter the cell genotype. For example, androgen activates androgen receptors (ARs) and promotes cell proliferation *via* altering gene expression (51). CRIF1 is involved in cancer cell proliferation *via* activating several critical signal transduction pathways, regulating hormone receptors or some transcriptional factors. This suggests that researchers could target CRIF1 to inhibit cancer cell proliferation through exercising its function of regulating cell proliferation.

CRIF1 is reported as a transcriptional coactivator of STAT3 and is important for maintaining STAT3 DNA binding activity (52). STAT3 is generally regarded as a direct transcription factor and also alters gene expression through causing epigenetic changes, such as DNA methylation and chromatin modulation. Many inhibitors targeting STAT3 are exploited for cancer treatment, including antisense oligonucleotides, STAT3 decoy oligonucleotides, and small-molecule inhibitors (53). Although the future of STAT3 inhibitors as therapies is promising, the toxicity is the most important side effect that has to be overcome. As a coactivator of STAT3, CRIF1 interacts with STAT3 *via* its C-terminal coiled-coil domain (CCD). STAT3 target genes such as Myc, Socs3, c-Fos, and JunB would be downregulated by CRIF1 deficiency. In mice with CRIF1 deficiency, both defective cellular proliferation and increased cell death would lead to embryonic growth deficit or death (52). Beyond this, CRIF1 negatively regulates phosphorylation of STAT3 *via* enhancing Socs3 activity in T cells (54). Taken together, CRIF1 is essential for STAT3 transcriptional activity. However, it also negatively regulates STAT3 phosphorylation. Exploiting new drugs targeting CRIF1 to interfere STAT3 DNA binding activity or enhancing CRIF1 activity to inhibit STAT3 phosphorylation are both potential strategies in cancer treatment.

Most of prostate cancers express AR throughout the course of the progression. Tan et al. revealed that CRIF1 plays a negative role in proliferation of prostate cancer cells (55). AR is a nuclear transcriptional factor regulated by ligand and drives cancer growth. AR signaling inhibitors (ARSIs) have been exploited and applied in clinical treatment for many years. However, the application of most ARSIs has been blighted by short-lived and therapy resistance (56). AR recruits CRIF1 to endogenous AR target promoters and interacts with either the N-terminal or C-terminal half-regions of CRIF1. Thus, the transactivation of AR is repressed. Interestingly, CRIF1 interacts with the C-terminal of AR and inhibits the interaction between p160 coactivator TIF2 and the AR C-terminal region. CRIF1 leads to AR N/C interaction disruption, and the activity of the AR N-terminal-DNA binding domain fragment decreased. CRIF1 outcompetes AR coactivators and represses AR transactivation. In addition, histone deacetylase 4 can be recruited by CRIF1 which also performs a negative role in transactivation of AR (55). Taken together, the CRIF1 potential acts as a natural inhibitor of AR and suppresses its DNA binding activity in prostate cancer. It is important to reveal the underlying effect of CRIF1 in AR-overexpressed cancers and try to fix the shortcomings of classic ARSIs.

Orphan nuclear receptor Nur77 is a transcription factor which is implicated in various biological processes like apoptosis and tumorigenesis (57–60). Recently, CRIF1 has been identified as a novel coregulator which interacts with Nur77 and participates in transactivation of Nur77. CRIF1 binds to the Nur77 AB domain and suppresses the transactivation of Nur77 which is mediated by TSH (61). Ultimately, CRIF1 suppresses the expression of Nur77 downstream genes and controls cell proliferation. In addition, CRIF1 function is inhibited by interacting with lymphocyte-specific protein tyrosine kinase (Lck) and leads to promotion of leukemic T-cell survival. Lck, which belongs to Src family kinases (SFKs), is universally expressed in T cells and easily detected in blood malignancies and many other solid tumors (62–64). In hepatocellular carcinoma (HCC), CRIF1 is beneficial to patient survival. These results show that CRIF1 suppresses epithelial–mesenchymal transition (EMT) *via* the TGFβ signaling pathway and inhibits the expression of matrix metalloproteinase-3 (MMP3) which plays an important role in tissue remodeling and metastasis (65). Although the direct role of CRIF1 in the proliferation of tumor cells has not been confirmed, it indeed participates in the proliferation of tumor cells through the interaction with STAT3, AR, NUR77, and other important cell proliferation regulation factors. Therefore, targeting CRIF1 can affect the activity of its binding factors, which is expected to go further in tumor treatment.

### The prospect of CRIF1 in cancer treatment

CRIF1 plays a critical role in cell cycle and cell proliferation in cancer and non-cancer cells. It regulates the cell cycle *via* directly binding to Gadd45 and CDK2. It also enhances P53 expression. In cell proliferation, CRIF1 always acts as a negative regulator in cancer cells. Although CDK inhibitors exhibit extraordinary capacity in anticancer, the toxicity, resistance, and off-target effect are ineluctable limitations encountered in clinical application. Recently, inhibitors targeting the CRIF1–CDK2 interface have exhibited a new strategy of indirectly suppressing CDK2 activity. Exploiting effective interface inhibitors are promising to try to overcome the shortcomings of other inhibitors encountered in clinical practice (8, 37). It provides an unprecedented strategy that targets CRIF1 rather than its binding proteins to overcome these shortcomings in radiotherapy and chemotherapy.

## CRIF1 in mitochondria-related diseases

### The discovery of CRIF1 in the mitoribosome

CRIF1 was originally identified only as a nuclear protein, due to binding to Gadd45 family proteins which locate in the nuclei. There is a nuclear localization sequence (NLS) in the C-terminal. To confirm the subcellular location of CRIF1, researchers constructed GFP tags exactly at its N-terminal (3). CRIF1 was then regarded as a nuclear protein for a long time. However, further studies found that CRIF1 could interact with LSUs and ICT1 near the polypeptide exit site of the mitoribosome (66–68). Due to the limitations of the LC-MS/MS method, CRIF1 was not detected as a mitoribosomal protein in the initial screening of mitoribosomal subunits (69, 70). Through protein sequence analysis, a mitochondrial targeting sequence (MTS) was predicted in the N-terminal region of CRIF1. Kim et al. identified that when GFP was tagged at the CRIF1 N-terminal region, it was only detected in the nuclei. In contrast, C-terminally tagged CRIF1 was mainly localized in mitochondria (15). This is because NLS is in the C-terminal region and MTS is in the N-terminal region. It suggests that CRIF1 might not only regulate the cell cycle in nuclei but also potentially regulate mitochondrial function.

**Figure 2:**
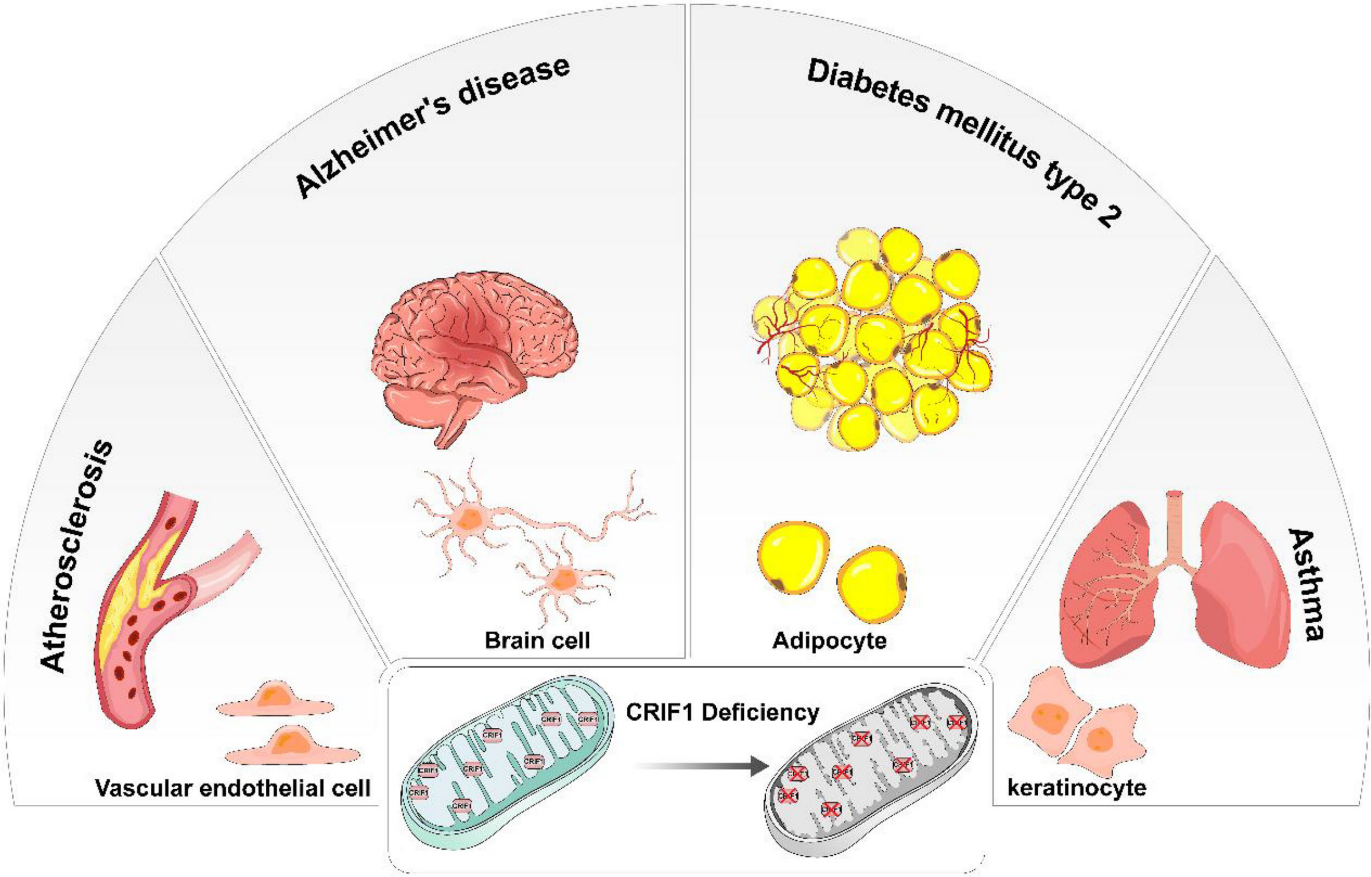
In different types of cells, CRIF1 deficiency induces mitochondrial dysfunction and eventually causes different diseases. In vascular endothelial cells, mitochondrial dysfunction induced by CRIF1 deficiency potentially causes atherosclerosis. In brain cells, mitochondrial dysfunction induced by CRIF1 deficiency potentially causes Alzheimer’s diseases. In adipocytes, mitochondrial dysfunction induced by CRIF1 deficiency potentially causes diabetes mellitus type 2. In keratinocytes, mitochondrial dysfunction induced by CRIF1 deficiency potentially causes asthma.

CRIF1 was cleaved after entering mitochondria and positioned close to the peptide exit tunnel of the mitoribosome and together with MRPL23, MRPL24, and MRPL44. Although mitoribosome biogenesis is not influenced by CRIF1, it indeed interacts with the LSU of the motoribosome and potentially acts as an integral component of the mitoribosome. Some molecular chaperones, especially Tid1, interact with CRIF1 and contributes to the translational and cooperative posttranslational integration of nascent OXPHOS subunits (14, 15). CRIF1 deficiency easily causes deficit of OXPHOS capacity and increases risk of diseases related mitochondrial dysfunction. Taken together, CRIF1 is an indispensable MRP in the mitoribosome. Its deficiency significantly causes aberrant insertion of nascent OXPHOS subunits and leads to defective intersubunit assembly and decreases translation activities of mitoribosomes associated with the inner mitochondrial membrane. Thus, CRIF1 deficiency has been implicated in aberrant energy metabolism and many diseases related to mitochondrial OXPHOS dysfunction such as vascular disorders, diabetes, and neurodegenerative diseases (**Figure 2**).

### CRIF1 in vascular endothelial disorders

Beyond energy supporting, the function of mitochondria in endothelial cells is signaling cellular responses to environmental cues (71). In endothelial cells, dysfunction of mitochondria is an important cause of cell senescence, angiogenesis, and adaptive responses to hypoxia, inflammation, and oxidative stress (72). CRIF1 is an indispensable component of MRP in the large subunit. Deficiency of CRIF1 easily causes mitochondrial dysfunction. It might ultimately lead to cardiovascular disorders (73).

CRIF1 deficiency would cause the downregulation of deacetylase SIRT3 and SIRT1 in endothelial cells. This is associated with endothelial inflammation and senescence. Senescence and inflammation of the vascular endothelium have been identified to easily induce vascular dysfunction (e.g., atherosclerosis and hypertension). SIRT3 is located in the mitochondrial matrix and regulates ROS production through promoting the expression of key mitochondrial metabolic and detoxification proteins (74–76). Due to lack of a stabilizing effect of CRIF1, the degradation of NRF2 and PGC1α was accelerated through proteasome-dependent ubiquitination and SIRT3 levels decreased (77). This changed the mitochondrial antioxidant mechanism and ultimately led to endothelial senescence. Similarly, downregulated SIRT1 by CRIF1 deficiency easily causes endothelial inflammation. SIRT1 was identified to exhibit an antagonistic cross talk with the NF-κB pathway and inhibit p65 translocation (78). CRIF1 deficiency downregulated SIRT1 levels and promoted transcription factor NF-κB translocation to the nucleus and decreased its inhibitor IκBα. The NF-κB pathway was then activated and continued to enhance proinflammatory cytokine expression and augment cellular inflammation (79). In a model of mice with endothelial CRIF1 deficiency, severe cardiac dysfunction and premature death could be rescued by injecting exogenously the SIRT1 activator (80). SIRT1 and SIRT3 are two important protection factors which could be downregulated by CRIF1 deficiency and potentially cause cardiovascular diseases. Thus, loss of CRIF1 expression in the mitochondria easily leads to endothelial cell dysfunction and exhibits cardiovascular diseases.

In addition, CRIF1 might also regulate endothelial function by influencing the folate cycle. Lee et al. revealed that CRIF1 deficiency promotes eNOS uncoupling by inhibiting the SIRT1-eNOS pathway and the *de novo* BH4 synthesis pathway, resulting in reduced NO production (81). The dihydrofolate reductase expression is reduced by CRIF1 deficiency and results in increased production of homocysteine (82). The reduced NO production and increased homocysteine production both contribute to endothelial impairment.

The redox p66shc is another targeted protein to be regulated by CRIF1 in endothelial cells. The redox p66shc, a member of the ShcA adaptor protein family, has been shown to regulate the oxidative function in endothelial cells, which contributes to endothelial dysfunction (83). Decreased CRIF1 stimulates p66shc expression, and upregulated p66shc further activates the generation of reactive oxygen species (ROS). As a result, mitophagy and endothelial activation are induced by p66shc-mediated ROS in endothelial cells (84, 85). However, the effect of mitophagy in vascular disorders is controversial and needs to be further studied. Collectively, many advances approved that CRIF1 always acts as a protective factor in vascular disorders. It is a promising target to reveal the underlying mechanism of vascular disorders with mitochondrial dysfunction.

### CRIF1 in metabolic diseases

Metabolic alteration potentially plays an important role in many pathological progresses. CRIF1, as a component of the mitoribosome, could be involved in regulating mitochondrial metabolism *via* various ways.

Adipose tissues support various aspects of metabolism; thus, adipogenesis is important for systematic metabolism. CRIF1 is identified to play an important role in adipogenic differentiation. In the bone marrow, adipocyte tissues derive from BMSCs. After irradiation, upregulated CRIF1 contributes to break the balance of adipogenic and osteogenic differentiation and BMSCs lean toward adipogenic differentiation (86). Mechanically, upregulated CRIF1 contributes to adipogenesis probably *via* the PKA-cAMP signaling pathway. The change in CRIF1 expression has no effect on PKAα cat level; however, these two proteins directly interact with each other and the downstream factors could be regulated (87). CRIF1 is involved in the regulation of adipocyte differentiation and development. Within CRIF1-deficient macrophages, impaired OXPHOS capacity leads to adipose inflammation and systematic insulin resistance (88). Moreover, CRIF1 deficiency partly affects the expression of several nuclear transcription factors such as PPARγ in adipose-derived stem cells (ADSCs). At the same time, the generation of endogenous PPARγ ligands might be influenced by CRIF1 (89). In other words, CRIF1 partly regulates adipogenic genes. In addition, different levels of CRIF1 deficiency show different results in adipocytes. Homozygotic loss of CRIF1 severely impairs the development of whit adipose tissue (WAT). Ultrastructural abnormalities of mitochondria such as swollen and distorted cristae are obvious in the adipocytes of WAT. Systematic inflammation which responds to CRIF1 deficiency is caused by macrophage infiltration. In contrast, heterozygous CRIF1 deficiency in adipocytes exhibits no difference from normal adipocytes except for OXPHOS capacity (90).

Many studies reported that the primary defects of mitochondrial electron transport chain activity protect from obesity and insulin resistance (91). CRIF1 deficiency easily induces defects of mitochondrial electron transport chain activity. Therefore, mice with CRIF1-deficient hepatocytes were used to reveal the mechanisms and factors underlying the changes in systemic energy (92). It is generally recognized that induced UPR^mt^ exerts beneficial effects on resistance of obesity and systematic metabolism (93, 94). Growth differential factor 15 (GDF15) and fibroblast growth factor 21 (FGF21) are two main mitokines which are responsive to UPR^mt^. Under mitochondrial stress in CRIF1-deficient cells, the level of GDF15 is increased *via* the p38–CHOP pathway which could regulate UPR^mt^. Consistent with the change in GDF15, the expression of angiopoietin-like 6 (ANGPTL6) is increased by the reduction in mitochondrial OXPHOS capacity. Moreover, ANGPTL6 increases the expression of PPARα *via* activating the ERK-MAPK pathway. PPARα plays a pivotal role in stimulating the secretion of FGF21 (95, 96). Both GDF15 and FGF21 confer metabolic benefits. Taken together, CRIF1 deficiency partly stimulates UPR^mt^ and enhances insulin sensitivity. It means that CRIF1 potential regulates insulin sensitivity and is involved in the progression of diabetes.

CRIF1 expression is also associated with diabetes. Kim et al. revealed that mitochondrial diabetes and progressive loss of beta cells were caused by disruption of CRIF1 in mouse islets (97). In hearing loss mice, which come from a mouse model of diabetes, an obvious reduction of CRIF1 could be easily detected in impaired mitochondria from the cochlea (98). Furthermore, some researchers hold the view that low-level and high-level mitochondrial dysfunction caused by CRIF1 deficiency might induce opposing metabolic effects. Severe mitochondrial dysfunction which is induced by CRIF1 homodeficiency in hypothalamic proopiomelanocortin (POMC) neurons unexpectedly leads to maturity-onset obesity, reduced energy expenditure, and glucose intolerance resembling features of human diabetes mellitus type 2 (DM2) in both male and female mice. However, mild mitochondrial dysfunction which acts as CRIF1 heterodeficiency promotes a high-turnover metabolism with enhanced thermogenesis and resistance to diet-induced obesity. Further research found that the level of Pomc transcription is upregulated by mitochondrial DNA-encoded peptide (MOTS-c) in coordination with STAT3. β-Endorphin, which potentially plays an indispensable role in mediating the communication between POMC neurons and distant adipose tissues, is markedly induced by CRIF1 heterodeficiency (99). This explains the controversial role of CRIF1 played before. In contrast to CRIF1 homodeficiency which leads to type 2 diabetes-like symptoms, mild CRIF1 deficit enhances metabolism and contributes to insulin sensitivity. Taken together, CRIF1 is partly involved in regulating adipocyte differentiation and energy metabolism changes. Besides, CRIF1 deficiency has implicated in metabolic disorder and contributes to diabetes.

### CRIF1 in other diseases

Many neurodegenerative diseases such as Alzheimer’s disease (AD) and Parkinson’s disease (PD) ubiquitously exhibit mitochondrial dysfunction (100, 101). Hence, the relationship between the expression of CRIF1, an indispensable component of mitochondria, and neurodegenerative diseases has aroused the interest of many researchers. As an irreversible neurodegenerative disorder with impaired cognition, AD is the most common form of dementia characterized by aberrant accumulation of amyloid beta (Aβ) peptide, tau neurofibrillary degeneration, microglial and astrocyte responses, and blood–brain barrier disruption (102). In sporadic AD, familial AD, and toxin-induced models, mitochondrial dysfunction can be easily confirmed (103). In line with this, CRIF1 expression is decreased in AD patients and AD mouse models. The accumulation of non-functional mitochondria contributes to Aβ peptide and tau pathologies, resulting in acceleration of the process of AD (104). Increasing CRIF1 expression significantly contributes to rescue Aβ-induced disruption of mitochondrial morphology. Mechanically, accumulation of Aβ mediated the increase in ROS production. Enhanced ROS and ROS-dependent sumoylation of transcription factor specificity protein 1 (SP1) mediates the reduction of CRIF1 in the pathological region of the brain (105). This suggests that CRIF1 potentially becomes a new therapeutic target to improve symptoms *via* cleaning Aβ. Similarly, the role of CRIF1 in the pathological process of other neurodegenerative diseases is also a valuable researching direction.

Due to CRIF1 deficiency, the differentiation of keratinocyte and hair follicle stem cells (HFSCs) is both impaired. Abnormal keratinocyte differentiation is perhaps related to compromising skin barrier and antimicrobial function, eventually resulting in epithelial dysregulation in asthma, eosinophilic esophagitis, and allergic rhinosinusitis (106). Mechanically, CRIF1 deficiency could also reduce epidermal proliferation and increase apoptosis, resulting in disrupted skin homeostasis. In addition, the hair growth cycle is significantly retarded *via* the Wnt/β-catenin signaling pathway which is downregulated by deletion of CRIF1 (107, 108).

A recent study shows that, disrupting the electron transport chain (ETC) by deleting CRIF1, mice exhibit upregulated resistance to sepsis. It can be a reason to explain why mitoribosome–target antibiotics have beneficial effects on disease tolerance which are irrelevant to their antibacterial activity (109). It suggests that CRIF1 is an important part of ETC and CRIF1 deficiency-caused ETC mild perturbation is beneficial to induce resistance of diseases such as sepsis. Moreover, CRIF1 could be a downstream target of lymphocyte expansion molecule (LEM) and benefit to activate cytotoxic CD8+ T cells (CTL) (110).

Although CRIF1 does not control mitoribosome biogenesis, it is indeed an indispensable component of large subunits of the mitoribosome. CRIF1 deficiency easily causes defects of OXPHOS capacity and leads to mitochondrial dysfunction. Many studies establish a mitochondrial dysfunction model by knocking down CRIF1 for investigating related diseases. CRIF1 deficiency occurs in many diseases, but there are no drugs or therapy targeting CRIF1 to try to cure these diseases. With the underlying mechanism of CRIF1 further developing, it is expected that exploiting drugs is based on fixing CRIF1 deficit to apply in treatment of diseases with mitochondrial dysfunction. Besides, Warburg proposed that persistent and irreversible injury of mitochondria is a key reason that turns normal cells into cancer cells (111). Moreover, cancer cells get energy depending on glycolysis, which has been identified as a biomarker of cancer progression (112, 113). Mitochondrial dysfunction has been identified to regulate normal cell or cancer cell growth *via* initiating several signaling pathways (114–116). Rapidly growing cancer cells can become glycolytic and be unable to upregulate OXPHOS indicating partial mitochondrial dysfunction (117–119). However, the role of CRIF1 playing between mitochondrial dysfunction and cancer cell proliferation is still unclear and needs further study.

## Conclusion and perspective

Since CRIF1 distributes both in cell nuclei and in mitochondria, it might regulate cancer cell progression through genomic and non-genomic ways. Moreover, CRIF1 plays a pivotal role in regulating OXPHOS capacity. Moreover, it can generally be regarded as a molecular target to establish a mitochondrial dysfunction model for studying mitochondria-related diseases. Whether CRIF1 affects mitochondrial energy supplication and simultaneously affects cell proliferation should be a further discussion. With the increasing obstacles encountered in cancer therapy, the studies of CRIF1 and its mechanism in regulating cell cycle and cell proliferation might be a promising direction of cancer therapy in the future. Moreover, exploiting therapies and drugs based on targeting CRIF1 exhibits considerable potential in cancer treatment. This review would provide a fascinating snapshot of many of these roles in cancer cell progression, targeted cancer therapy, and treatment of diseases related to mitochondrial dysfunction, providing a more comprehensive holistic view of CRIF1.

## Author contributions

ZL and QR contributed to conception and design of the review. YJ and YX wrote the first draft of the manuscript. WZ, ZY and LX wrote sections of the manuscript. CL drew the figure. YNX and LC revised the manuscript. All authors contributed to manuscript revision, read and approved the submitted version.

## Funding

This work was supported by projects of the International Cooperation and Exchanges NSFC (No. 82020108025), National Natural Science Foundation of China (No. 81972973), and Chongqing Science and Technology Commission (cstc2021jcyj-jqX0004).

## Conflict of interest

The authors declare that the research was conducted in the absence of any commercial or financial relationships that could be construed as a potential conflict of interest.

## Publisher’s note

All claims expressed in this article are solely those of the authors and do not necessarily represent those of their affiliated organizations, or those of the publisher, the editors and the reviewers. Any product that may be evaluated in this article, or claim that may be made by its manufacturer, is not guaranteed or endorsed by the publisher.
