# Corrigendum: Multifunctions of CRIF1 in cancers and mitochondrial dysfunction

**DOI:** 10.3389/fonc.2023.1271492

**Published:** 2023-08-24

**Authors:** Yangzhou Jiang, Yang Xiang, Chuanchuan Lin, Weiwei Zhang, Zhenxing Yang, Lixin Xiang, Yanni Xiao, Li Chen, Qian Ran, Zhongjun Li

**Affiliations:** ^1^ Laboratory of Radiation Biology, Laboratory Medicine Center, Department of Blood Transfusion, The Second Affiliated Hospital, Army Military Medical University, Chongqing, China; ^2^ State Key Laboratory of Trauma, Burn and Combined Injuries, The Second Affiliated Hospital, Army Medical University, Chongqing, China

**Keywords:** CR6-interacting factor 1, cell cycle regulation, cell proliferation regulation, cancer treatment, mitochondrial ribosomal protein, mitochondrial dysfunction

In the published article, there was an error in the Funding statement. The number for the funding from Chongqing Science and Technology Commission was incorrectly displayed as “No. 2021C035”, the correct number is “cstc2021jcyj-jqX0004”. The correct Funding statement appears below.

**FUNDING**

This work was supported by projects of the International Cooperation and Exchanges NSFC (No. 82020108025), National Natural Science Foundation of China (No. 81972973), and Chongqing Science and Technology Commission (cstc2021jcyj-jqX0004).

The authors apologize for this error and state that this does not change the scientific conclusions of the article in any way. The original article has been updated.

